# Predicting Leptospirosis Using Baseline Laboratory Tests and Geospatial Mapping of Acute Febrile Illness Cases Through Machine Learning-Based Algorithm

**DOI:** 10.7759/cureus.73779

**Published:** 2024-11-15

**Authors:** Mallika Sengupta, Aditya Kundu, Saikat Mandal, Shiv Sekhar Chatterjee, Ujjala Ghoshal, Sayantan Banerjee, Kaushik Mukhopadhyay

**Affiliations:** 1 Microbiology, All India Institute of Medical Sciences, Kalyani, IND; 2 General Internal Medicine, All India Institute of Medical Sciences, Kalyani, IND; 3 Pharmacology, All India Institute of Medical Sciences, Kalyani, IND

**Keywords:** acute febrile illness, fever, k-nearest neighbours, leptospira, machine learning model

## Abstract

Introduction

Leptospirosis is a zoonotic infection caused by *Leptospira* bacteria, which is reemerging in various regions and often poses a diagnostic challenge due to its nonspecific symptoms. While most infections are mild, severe cases occur in 5-10% of patients and are associated with high mortality, especially in areas with poor sanitation and urbanization. This study aims to investigate the association of specific parameters with leptospirosis diagnosis using a machine learning model and geographic mapping tools to identify spatial patterns and high-risk areas for the disease.

Methods

An observational retrospective study conducted at a tertiary care center analyzed patients clinically suspected of leptospirosis over the course of one year. The study utilized laboratory investigations, geographic mapping, and machine learning models to explore the association between various laboratory parameters and the predictive diagnosis of leptospirosis.

Results

The study, conducted over one year at All India Institute of Medical Sciences, Kalyani, India, included 325 patients, of whom 43 (13.2%) tested positive for leptospirosis by IgM ELISA. Geographic mapping revealed case clusters around nearby districts of West Bengal, with a few cases from Tripura and Bangladesh. The study found no significant association between individual laboratory parameters and leptospirosis diagnosis. However, machine learning models, particularly k-nearest neighbors (KNN), demonstrated moderate predictive accuracy (accuracy: 74%, area under the curve: 0.6).

Conclusion

Geographic mapping identified clusters of leptospirosis cases; however, no significant association was found between individual laboratory parameters and the disease diagnosis. Machine learning models, particularly KNN, demonstrated moderate predictive accuracy. The study also highlighted the overlapping clinical features of leptospirosis, dengue, and scrub typhus in West Bengal, although it noted the absence of detailed clinical data as a limitation.

## Introduction

Leptospirosis, a zoonotic disease caused mainly by pathogenic species of *Leptospira*, is reemerging in countries such as China, Japan, Australia, India, and Europe [1]. While most infections are subclinical or mild, severe disease occurs in 5-10% of patients, with high mortality rates [2]. The illness is often difficult to differentiate from other acute febrile illnesses, such as scrub typhus, dengue, and malaria, due to its nonspecific symptoms, including fever, headache, abdominal pain, myalgia, and conjunctival suffusion [3]. Leptospirosis is a global disease affecting both humans and animals, caused by the spirochete bacterium *Leptospira*, which is carried by animals and spread through contaminated water and soil, typically due to animal waste. It is often neglected and reemerging in developing countries, exacerbated by poor sanitation and urbanization. The disease can cause symptoms ranging from mild fever to severe organ damage, with some rare and unusual symptoms also reported [4].

In 90% of cases, the infection is self-limiting, with an incubation period of 10 days on average (typically ranging from five to 14 days). Classic cases progress through two phases: an initial septicemic phase followed by an immune phase [5]. The illness often begins with a high fever (38-40 °C), accompanied by headaches, chills, rigors, and muscle pain. During the septicemic phase, additional symptoms may include abdominal pain, loss of appetite, nausea, vomiting, diarrhea, cough, and sore throat. Although less common, physical signs like conjunctival redness and tenderness in the calf and lower back muscles may also be observed. A maculopapular rash is rare. Upon examination, swollen lymph nodes, an enlarged spleen, and liver enlargement may be noted. This acute phase typically lasts five to seven days. Given the nonspecific nature of these symptoms, leptospirosis must be distinguished from illnesses such as malaria, dengue, and rickettsial fever [6]. In the immune phase, the organism is eliminated from the bloodstream but can still be detected in tissues and urine. Along with symptoms from the acute phase, the immune phase may include jaundice, kidney failure, irregular heart rhythms, respiratory symptoms, aseptic meningitis, conjunctival redness, and sensitivity to light [5].

Although culture is considered the gold standard for diagnosing leptospirosis, it cannot be performed on all samples. Thus, composite diagnostic criteria are necessary to ensure no cases are missed. Frameworks such as those from the WHO, CDC, and Faine’s criteria (and its modification by Shivakumar and Shareek in Chennai) provide such a framework [7]. However, these definitions can be impractical, as culture and PCR have low sensitivity, and obtaining paired serum samples from patients with acute febrile illnesses like leptospirosis is challenging, especially in resource-limited settings. Additionally, a rise in antibody titers may not be detectable if the patient has received antimicrobial drugs during the illness. The modified Faine’s criteria, along with the CDC and WHO case definitions, focus primarily on clinical features and specific laboratory tests for leptospirosis, excluding other routine laboratory investigations, such as complete blood count, liver function tests, and kidney function tests, which may provide valuable diagnostic information.

This study aims to evaluate the potential of basic laboratory parameters in predicting leptospirosis using various machine learning models and geographic mapping tools to analyze the spatial distribution of leptospirosis cases in a tertiary care center in eastern India. Through this dual approach, we seek to enhance diagnostic capabilities for leptospirosis beyond traditional criteria and gain insights into its geographical distribution, thereby improving disease surveillance and control measures.

## Materials and methods

This observational retrospective study was conducted in the Department of Microbiology at All India Institute of Medical Sciences (AIIMS), Kalyani, India, over a one-year period, from January 1, 2023 to December 31, 2023. The study received approval from the Institutional Ethics Committee of AIIMS, Kalyani (approval number IEC/AIIMS/Kalyani/certificate/2024/041). The ethical approval was in accordance with the ethical standards of the responsible committee on human experimentation, adhering to the principles outlined in the Helsinki Declaration. Due to the study’s retrospective nature and the exclusive use of laboratory data, the ethics committee granted a waiver of informed consent.

Study population and data collection

The study included all patients clinically suspected of leptospirosis, regardless of their test results. For each patient, a comprehensive panel of laboratory investigations was recorded, which included complete blood count, liver function tests, kidney function tests, and leptospirosis IgM ELISA results. Additionally, test results for other febrile illnesses were documented, including malaria (diagnosed by microscopy and antigen detection), dengue (NS1 and IgM ELISA), enteric fever serology, blood culture, and scrub typhus IgM ELISA. The ELISA test for leptospirosis was performed following the manufacturer’s instructions, with all results carefully documented.

Geographical mapping

To elucidate spatial patterns and identify high-risk areas for leptospirosis, geographical mapping of positive cases was performed using Google Earth (Google LLC, Mountain View, California, USA). This approach allowed for the visualization of case distribution and potential clustering, offering valuable insights into the epidemiological landscape of the disease.

Statistical analysis and data visualization

Data management and statistical analyses were conducted using the R programming language. The compareGroups and tidyverse libraries were utilized for comprehensive statistical analysis and advanced data visualization. These tools facilitated the exploration of associations between various laboratory parameters and leptospirosis diagnosis. A p-value of less than 0.05 was considered statistically significant.

Machine learning model

To determine the predictive diagnosis of leptospirosis from basic laboratory parameters, a suite of machine learning models was implemented using the TensorFlow library in Python. The following algorithms were employed: k-nearest neighbors (KNN), AdaBoost, decision tree, Naive Bayes, random forest, logistic regression, and support vector machine (SVM). These models were trained on the collected laboratory data, with the dataset appropriately split into training and testing sets to assess model performance.

Performance metrics

The efficacy of each machine learning model was assessed using a comprehensive set of performance metrics, including accuracy, sensitivity, specificity, positive predictive value (PPV), negative predictive value (NPV), and the area under the receiver operating characteristic curve (AUC-ROC). Accuracy quantified the overall correctness of predictions, while sensitivity and specificity measured the models’ ability to correctly identify positive and negative cases, respectively. PPV and NPV provided insights into the reliability of positive and negative predictions. The AUC-ROC served as a robust measure of each model’s discriminative capability, with values ranging from 0 to 1, where 1 indicates perfect classification.

## Results

**Figure 1:**
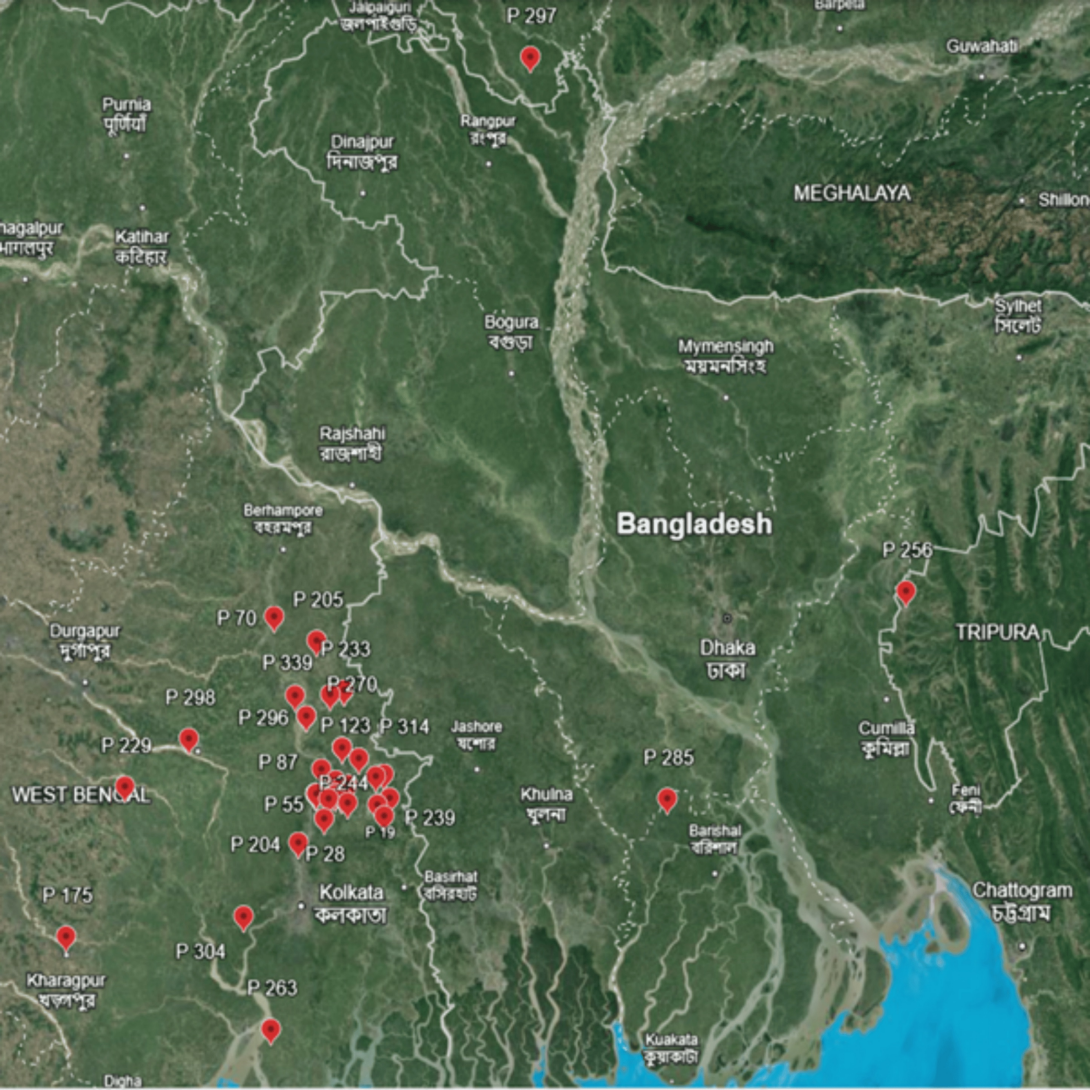
Geospatial mapping of Leptospira-positive cases in Nadia, West Bengal, and surrounding regions Red markers indicate the locations of positive cases reported in this study (map created using Google Earth, Google LLC, Mountain View, California, USA)

A retrospective observational study was conducted in the Department of Microbiology at AIIMS, Kalyani, India, over one year (January to December 2023). The study included 325 patients, of whom 43 (13.2%) tested positive for leptospirosis by IgM ELISA. Geographic mapping of all positive leptospirosis cases was performed using Google Earth, revealing that most cases clustered around the center, particularly in the districts of Nadia, 24 Parganas North, 24 Parganas South, Kolkata, and Hooghly in West Bengal. Additionally, a few cases were reported from Tripura (P256) and Bangladesh (P285 and P297), as shown in Figure 1.

The population characteristics of all positive and negative cases of leptospirosis, including gender and basic laboratory tests (total lymphocyte count, platelet count, hemoglobin, liver function tests such as total bilirubin, direct bilirubin, aspartate aminotransferase, alkaline phosphatase, kidney function tests like urea and creatinine), are presented in Table 1. Among the included cases, none were positive for *Salmonella* Typhi serology or malaria. However, one patient had a blood culture positive for *Escherichia coli*. Additionally, 20 (6.15%) patients were positive for scrub typhus by IgM ELISA, and 33 (10.2%) were positive for dengue by NS1 and IgM ELISA. This emphasizes that leptospirosis, dengue, and scrub typhus are the most significant causes of acute febrile illness with overlapping clinical features, particularly in the eastern part of India. 

**Table 1:** Population characteristics and laboratory parameters of suspected Leptospirosis cases ALP, alkaline phosphatase; AST, aspartate aminotransferase

Characteristic	Value	N
Age, mean (SD)	35.9 (16.3)	325
Gender, n (%)		325
Female	174 (53.5%)	
Male	151 (46.5%)	
*Leptospira* status, n (%)		325
Negative	282 (86.8%)	
Positive	43 (13.2%)	
Total lymphocyte count, mean (SD)	7.76 (4.49)	325
Neutrophil (%), mean (SD)	63.1 (16.0)	324
Lymphocyte (%), mean (SD)	27.5 (13.8)	324
Platelet count, mean (SD)	226 (107)	325
Hemoglobin (g/dL), mean (SD)	12.0 (10.3)	325
Total bilirubin (mg/dL), mean (SD)	1.19 (3.47)	298
Direct bilirubin (mg/dL), mean (SD)	0.68 (3.07)	298
AST (U/L), mean (SD)	89.6 (212)	298
ALP (U/L), mean (SD)	86.7 (241)	298
Urea (mg/dL), mean (SD)	23.2 (16.2)	286
Creatinine (mg/dL), mean (SD)	0.81 (0.51)	289
CRP (mg/L), mean (SD)	47.5 (74.4)	248
Malaria smear and antigen test, n (%)	0 (0%)	325
*Salmonella* Typhi serology, n (%)	0 (0%)	325
Scrub typhus IgM detection, n (%)	20 (6.15%)	325
Blood culture, n (%)	1 (0.31%)	325
Dengue NS1 and IgM detection, n (%)	33 (10.2%)	325

**Figure 2:**
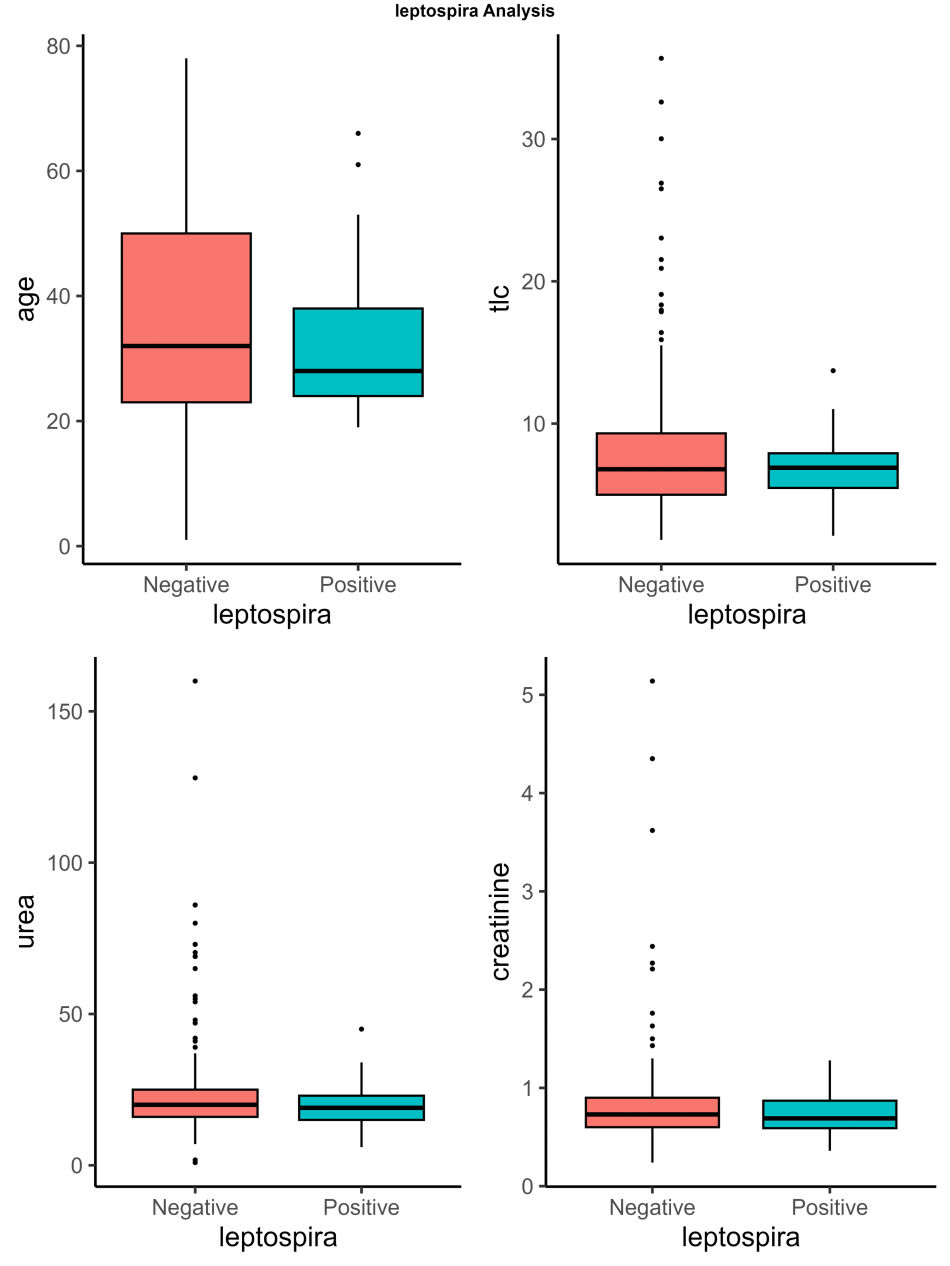
Box plots illustrating the distribution of key laboratory parameters in leptospirosis-positive and leptospirosis-negative cases

Additionally, one case was found to be positive for both leptospirosis and scrub typhus, while three cases were positive for both leptospirosis and dengue serology. Table 2 presents the association between various laboratory parameters and the diagnosis of leptospirosis. It was observed that no individual laboratory parameter was significantly associated with the diagnosis of leptospirosis (Figure 2).

**Table 2:** Association of laboratory parameters between Leptospira-positive and Leptospira-negative cases Data are presented as mean ± SD for continuous variables and n (%) for categorical variables. Continuous variables were compared using independent t-tests, and categorical variables were compared using the chi-square test or Fisher’s exact test as appropriate. * p < 0.05 indicates statistical significance. ALP, alkaline phosphatase; AST, aspartate aminotransferase; TLC, total leukocyte count

Characteristic	Negative (N = 282)	Positive (N = 43)	Test statistic	p-value
Demographics				
Age (years)	36.4 ± 16.9	32.0 ± 11.4	t = 2.16	0.032*
Gender				
Female	146 (51.8%)	28 (65.1%)	χ² = 2.16	0.142
Male	136 (48.2%)	15 (34.9%)		
Laboratory parameters				
TLC (×10³/μL)	7.91 ± 4.72	6.80 ± 2.29	t = 2.45	0.015*
Neutrophil (%)	63.6 ± 16.0	60.3 ± 16.0	t = 1.23	0.221
Lymphocyte (%)	27.1 ± 13.8	30.1 ± 13.9	t = -1.30	0.196
Platelet (×10³/μL)	224 ± 107	239 ± 108	t = -0.85	0.396
Hemoglobin (g/dL)	12.0 ± 11.1	11.6 ± 2.02	t = 0.53	0.596
Total bilirubin (mg/dL)	1.05 ± 2.96	2.14 ± 5.78	t = -1.14	0.257
Direct bilirubin (mg/dL)	0.56 ± 2.60	1.48 ± 5.20	t = -1.08	0.283
AST (U/L)	86.0 ± 205	113 ± 253	t = -0.64	0.521
ALP (U/L)	87.5 ± 254	81.2 ± 123	t = 0.25	0.804
Urea (mg/dL)	23.6 ± 17.1	20.1 ± 8.05	t = 2.01	0.045*
Creatinine (mg/dL)	0.82 ± 0.54	0.73 ± 0.19	t = 2.11	0.036*
CRP (mg/L)	48.6 ± 76.8	39.4 ± 53.6	t = 0.83	0.41
Serological tests				
Malaria	0 (0%)	0 (0%)	-	-
*Salmonella*Typhi	0 (0%)	0 (0%)	-	-
Scrub typhus	19 (6.74%)	1 (2.33%)	Fisher’s exact	0.492
Blood culture	1 (0.35%)	0 (0.00%)	Fisher’s exact	1
Dengue	30 (10.6%)	3 (6.98%)	Fisher’s exact	0.594

Seven machine learning models were evaluated for predicting leptospirosis using basic laboratory parameters (Table 3). The KNN algorithm exhibited the highest discriminative ability, with an AUC of 0.6, accuracy of 0.74, sensitivity of 0.28, and specificity of 0.81. AdaBoost achieved the highest accuracy (0.76) but had a lower sensitivity (0.1). The Naive Bayes model demonstrated the highest sensitivity (0.37) among all models but showed the lowest accuracy (0.49). Random forest, logistic regression, and SVM models failed to identify any positive cases, as evidenced by their sensitivity of 0.

**Table 3:** Performance of machine learning models in predicting leptospirosis from test data AUC, area under the curve; KNN, K-nearest neighbors; NPV, negative predictive value; PPV, positive predictive value; SVM, support vector machine

Model	Accuracy	Sensitivity	Specificity	PPV	NPV	AUC
KNN	0.74	0.28	0.81	0.18	0.88	0.6
AdaBoost	0.76	0.1	0.86	0.1	0.86	0.51
Decision tree	0.68	0.19	0.75	0.1	0.86	0.48
Naive Bayes	0.49	0.37	0.51	0.11	0.84	0.45
Random forest	0.79	0	0.91	0	0.86	0.4
Logistic regression	0.68	0	0.78	0	0.84	0.3
SVM	0.83	0	0.96	0	0.87	0.3

**Figure 3:**
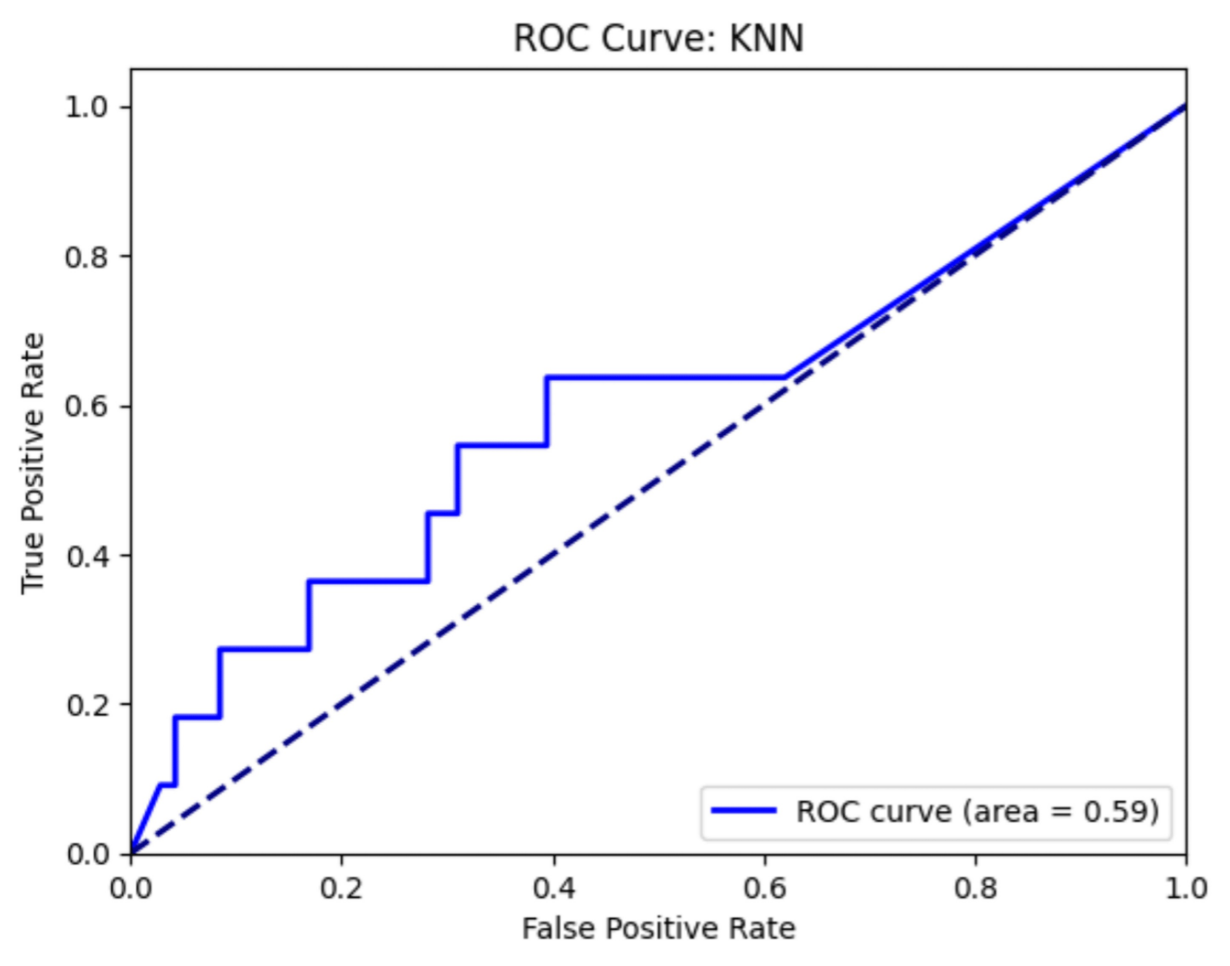
ROC curve for the best model, i.e., KNN model KNN, k-nearest neighbors; ROC, receiver operating characteristic

All models demonstrated high NPVs, ranging from 0.84 to 0.88, but consistently low PPVs, between 0 and 0.18 (Figure 3). Additionally, no significant association was observed between individual laboratory parameters and the diagnosis of leptospirosis.

## Discussion

Leptospirosis is a zoonotic disease, with the infection maintained in the kidneys of reservoir animals such as rodents, cattle, goats, and pigs. A systematic review of leptospirosis in India found that most cases were reported from the coastal belt (Gujarat, Tamil Nadu, Maharashtra, Kerala, Karnataka, and the Andaman and Nicobar Islands) [8]. However, this study highlights the presence of leptospirosis in West Bengal. The review attributes the increased incidence of leptospirosis in southern coastal areas to factors such as high population density near rivers, heavy rainfall, and warm, humid environments [8]. These same factors are prevalent in the districts of Nadia, 24 Parganas North, 24 Parganas South, Kolkata, and Hooghly in West Bengal, contributing to the high number of leptospirosis cases in the region.

Leptospirosis in humans presents with a wide range of clinical symptoms, from mild, self-limiting fever to severe, life-threatening conditions affecting multiple organs. Various organ systems can be involved, and atypical complications are also reported. The clinical signs of leptospirosis often resemble other tropical febrile illnesses, including dengue, and rickettsial infections such as scrub typhus, bacterial sepsis, and malaria [9]. Both scrub typhus and leptospirosis share nonspecific clinical symptoms, making it difficult to distinguish them based on clinical signs alone. Many diagnostic studies for these infections have relied on serological tests, raising the possibility that observed co-infections could be due to cross-reactivity in serology or the emergence of a new infection in someone previously exposed to the other pathogen. As noted by Gupta et al., it is also possible that many past diagnoses of leptospirosis were actually cases of scrub typhus, and vice versa [10]. A study investigating the co-infection of leptospirosis and scrub typhus in febrile patients found that, of 26 samples with IgM antibodies to Leptospira, 11 also had IgM antibodies to scrub typhus [11]. In our study, one patient was diagnosed with both scrub typhus and leptospirosis.

Dengue and leptospirosis coinfections pose diagnostic challenges due to their overlapping clinical manifestations, which are common in tropical and subtropical regions. In a case series, two Bangladeshi males, aged 25 and 35, presented with acute febrile illnesses initially suspected to be dengue based on serological tests. One patient experienced hepatic and renal involvement, while the other exhibited symptoms resembling meningoencephalitis, complicating the clinical diagnosis. Upon further investigation, Leptospira coinfection was identified, and both patients received targeted treatment for dengue along with antibacterial therapy for leptospirosis, resulting in their recovery [12]. In our study, three patients were diagnosed with both dengue and leptospirosis concurrently.

The use of deep learning and machine learning in medical science is rapidly expanding, particularly in the analysis of visual, audio, and language data. In one study, an optimized ensemble model combining a deep neural network (DNN) with two machine learning models was developed to predict diseases based on laboratory test results. The study utilized 86 laboratory attributes from 5,145 cases spanning 39 diseases to build LightGBM, XGBoost, and DNN models, achieving a prediction accuracy of 92% for the most common diseases. Feature importance was analyzed using SHAP values, demonstrating high efficiency in disease prediction [13].

A retrospective model development and validation study created a deep learning algorithm to differentiate Kawasaki disease from multisystem inflammatory syndrome in children. The model incorporated patient age, the five classic clinical signs of Kawasaki disease, and 17 laboratory measurements. Multisystem inflammatory syndrome is a newly identified condition that emerged during the COVID-19 pandemic, characterized by widespread inflammation occurring following a SARS-CoV-2 infection [14].

Another study highlights the growing role of machine learning in diagnosing dengue in patients presenting with acute febrile illness. By using supervised machine learning models, specifically XGBoost, the study demonstrated the model's ability to differentiate dengue from other febrile illnesses based on early clinical data, achieving an AUROC of 0.86. The study also emphasized the influence of seasonality on model performance and suggested the use of dynamic thresholds to maintain diagnostic consistency year-round, which could improve patient care and public health interventions in dengue-endemic regions [15].

However, the machine learning model’s performance in predicting leptospirosis based on laboratory parameters was suboptimal, with even the best-performing model (KNN) showing only marginal improvement over random chance. This limited predictive power is likely due to the complex presentation of leptospirosis and the multifactorial nature of its diagnosis.

The study’s limitations include the absence of detailed clinical data. Relying solely on laboratory parameters, it lacked comprehensive clinical information such as patient history, symptoms, and physical examination findings, which likely reduced the models’ ability to differentiate leptospirosis from other febrile illnesses with overlapping clinical features. Furthermore, the low predictive accuracy of the machine learning models, especially KNN, was a limitation. With an AUC of 0.6, the models demonstrated moderate accuracy and exhibited low sensitivity and PPV, suggesting a high rate of false positives. The study was conducted at a single tertiary care center in eastern India, limiting the generalizability of its findings, as the sample size may not represent the broader population affected by leptospirosis. Lastly, the overlapping clinical and serological profiles of leptospirosis with diseases like dengue and scrub typhus complicated the identification of co-infections, potentially affecting the model’s performance.

Future studies aim to incorporate comprehensive clinical data, expand the geographic scope, and include advanced diagnostic markers to improve model accuracy and clinical applicability. This holistic approach may enhance model performance and lead to the development of more reliable diagnostic tools for leptospirosis. Additionally, exploring advanced machine learning techniques and larger, more diverse datasets could further improve predictive capabilities in this challenging diagnostic landscape.

## Conclusions

Geographic mapping successfully identified clusters of leptospirosis cases, primarily in districts of West Bengal. Despite no significant association between individual laboratory parameters and leptospirosis diagnosis, machine learning models, particularly KNN, demonstrated a moderate predictive accuracy of 60%. The study emphasized the clinical overlap between leptospirosis, dengue, and scrub typhus, with the absence of detailed clinical data identified as a key limitation. Future prospective studies should incorporate comprehensive clinical data and refine machine learning models to enhance predictive accuracy for leptospirosis while addressing the diagnostic challenges posed by the overlap with other febrile illnesses such as dengue and scrub typhus.
